# SUBCLAVIAN TO AORTA BYPASS FOR ADULT AORTIC COARCTATION

**Published:** 2010

**Authors:** Barbaros Kinoglu, Faruk Hokenek, Murat Ugurlucan, Levent Kaplan

**Affiliations:** *Medical Park Hospital, Cardiovascular Surgery Clinic, Fatih, Istanbul, Turkey*

The patient was a 42-year-old male who presented to the clinic with headache and back pain. Physical examination of the chest was completely normal. There was significant pulse volume difference between upper and lower exremities on both sides. There was 60mmHg systolic blood pressure gradient between upper and lower extremities. Aortic pathology was considered and multislice computerized tomography angiography of thoroaco-abdominal aorta was performed.

**Fig 1:**
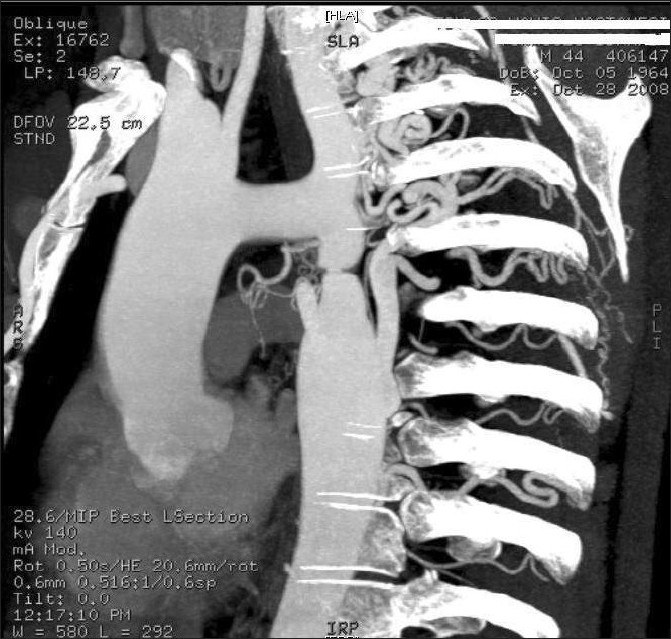


**Fig 2:**
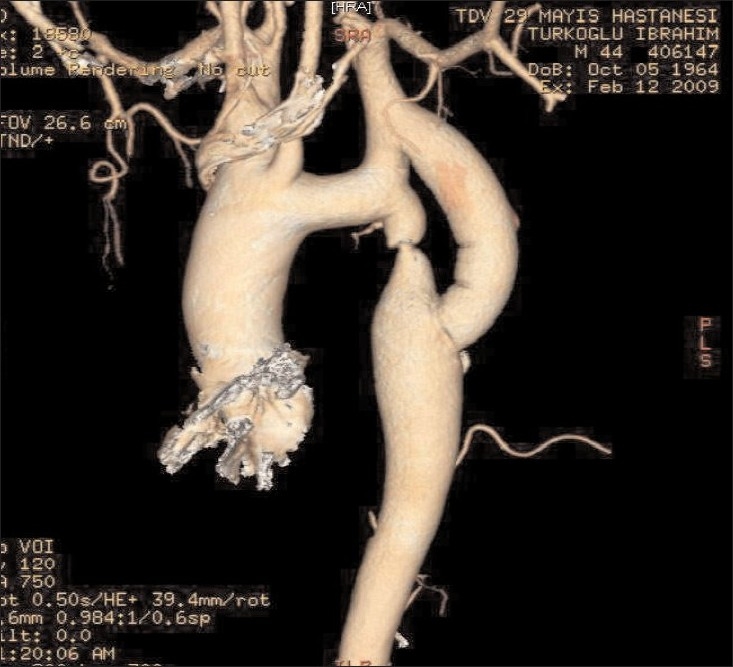


The diagnosis was aortic coarction (Figure 1). The patient underwent surgical treatment with left subclavian artery to descending aorta bypass with an 18mm Dacron tube graft (Figure 2).

Aortic coarctation is a well known congenital cardiac disease which is generally treated during childhood. The patient refused angioplaty. There are various surgical techniques for the treatment of aortic coarctation and left subclavian artery to descending aorta extra-anatomical bypass procedure is among these reliable methods with successfull outcome^1^–^3^.
